# EXPLORING THE LANDSCAPE OF GENERATIVE AI AND LARGE LANGUAGE MODELS IN ALZHEIMER AND DEMENTIAS RESEARCH

**DOI:** 10.1093/geroni/igae098.2976

**Published:** 2024-12-31

**Authors:** Duo Wei, Jeannine Elmasri, Bianca Hernandez, Riya Goyal, David Burdick

**Affiliations:** Stockton University, Galloway, New Jersey, United States; Stockton University, Galloway, New Jersey, United States; New Jersey Institute of Technology, Newark, New Jersey, United States; Stockton University, Galloway, New Jersey, United States; Stockton University, Galloway, New Jersey, United States

## Abstract

With the introduction of ChatGPT in November 2022 and Google Bard (now Gemini) in March 2023, the artificial intelligence (AI) landscape has undergone a significant and rapid evolution. An in-depth scoping review was utilized to investigate the application of generative AI and Large Language Models (LLM) in research on Alzheimer’s Disease and Related Dementias (ADRD) among older adults. The objectives were twofold: first, to investigate specific applications of generative AI and LLM in ADRD research, and second, to delineate the diverse usage of these technologies within this domain. Leveraging PubMed, search terms such as “AI,” “Dementia/Alzheimer,” and “Older Adults” were employed, resulting in the retrieval of 196 articles published in January 2022-December 2023. Of 196 articles found, 145 meeting eligibility criteria were meticulously categorized into two topics: ADRD domain and AI-related tasks. Statistical analysis revealed compelling insights, with significant differences in the application of generative AI (χ2 = 21.91, p <.005) and LLM (χ2 = 21.42, p <.005) in ADRD research. Notably, generative AI predominantly focused on “Research/Drug Discovery” (34.38%) and “Improved Treatment & Care” (37.50%), while LLM emphasized “Challenges/Opportunities” and “Early Detection/Diagnosis.” Within the ADRD domain, articles were categorized into distinct areas such as “Care,” “Cognitive Decline,” “Diagnosis,” “Genetics,” “Imaging,” and “Other.” Caregiving and support emerged as predominant themes, comprising 55% of the reviewed articles, followed by diagnosis (34%). This comprehensive analysis sheds light on the multifaceted applications of AI in ADRD research, offering valuable insights into potential areas for further investigation.

